# The parietal cell gastric H, K-ATPase also functions as the Na, K-ATPase and Ca-ATPase in altered states

**DOI:** 10.12688/f1000research.2-165.v2

**Published:** 2013-09-12

**Authors:** Tushar Ray

**Affiliations:** 1 Ramakrishna Vedanta Ashrama of Phoenix, Tempe, AZ 85281, USA; 2 SUNY Upstate Medical Center, Syracuse, NY 13210, USA

## Abstract

This article offers an explanation for the apparent lack of Na, K-ATPase activity in parietal cells although ouabain has been known to inhibit gastric acid secretion since 1962. The gastric H, K-ATPase (proton-pump) seems to be acting in altered states, thus behaving like a Na, K-ATPase (Na-pump) and/or Ca-ATPase (Ca-pump) depending on cellular needs.  This conclusion is based on the following findings. First, parietal cell fractions do not exhibit Na, K-ATPase activity at pH 7.0 but do at pH 8.5. Second, the apical plasma membrane (APM) fraction exhibits a (Ca or Mg)-ATPase activity with negligible H, K-ATPase activity. However, when assayed with Mg alone in presence of the 80 k Da cytosolic proton-pump activator (HAF), the APM fraction reveals remarkably high H, K-ATPase activity, suggesting the observed low affinity of Ca (or Mg)-ATPase is an altered state of the latter. Third, calcium (between 1 and 4 µM) shows both stimulation and inhibition of the HAF-stimulated H, K-ATPase depending on its concentration, revealing a close interaction between the  proton-pump activator and local Ca concentration in gastric H, K-ATPase function. Such interactions suggest that Ca is acting as a terminal member of the intracellular signaling system for the HAF-regulated proton-pump. It appears that during resting state, the HAF-associated H, K-ATPase remains inhibited by Ca (>1 µM) and, prior to resumption of acid secretion the gastric H, K-ATPase acts temporarily as a Ca-pump for removing excess Ca from its immediate environment. This conclusion is consistent with the recent reports of immunochemical co-localization of the gastric H, K-ATPase and Ca-ATPase by superimposition in parietal cells, and a transitory efflux of Ca immediately preceding the onset of acid secretion. These new perspectives on proton-pump function would open new avenues for a fuller understanding of the intracellular regulation of the ubiquitous Na-pump.

## Introduction

At the peak of acid secretion gastric juice has a pH close to 0.1 compared to blood (pH, 7.4). Based on this the parietal cells transport protons against a concentration gradient of over a million fold mediated by the gastric H, K-ATPase system. This member of the P-2 ATPase family has been the most extensively studied along with the Na, K-ATPase and Ca-ATPase families due to their prominent roles in health and disease. Major developments in the field occurred following the single topology scheme for the Na, K-ATPase reaction proposed by Post and Albers in the early 1960s, which was subsequently extended to the H, K-ATPase system. The Post-Albers (PA) scheme visualizes Na-dependent phosphorylation of the 100 k Da α-subunit by ATP (a kinase step) and a sequential K-dependent dephosphorylation (a phosphatase step) during each reaction cycle. The activity of K-dependent p-nitrophenyl phosphatase (K-pNPPase), which is always co-purified with the Na, K-ATPase system was assumed to represent the phosphatase step of the total ATPase reaction. However this assumption was subsequently proven to be erroneous since K-pNPPase activity reflects the ion channel activity across the membrane rather than being a partial reaction of the ATPase ^1^. Based on the orientation of the ATP hydrolytic sites and the associated pNPPase sites together with their corresponding K regulatory sites (of high and low affinity respectively) across the membrane ^1, 2^ a mirror image orientation of the two α-subunits (within the functional H, K-ATPase complex) was proposed. The dual topology model fits well with numerous reports in the literature ^3^.

This unified dual topology model helped to clarify the demonstrated lack of Na, K-ATPase activity in parietal cell fractions so far using the conventional assay procedure even though ouabain (a Na-pump inhibitor) has long been known to eliminate gastric acid secretion ^4^. However, gastric H, K-ATPase activity shows stimulation ^5^ by sodium manifesting a Na, K-ATPase activity when assayed at pH 8.5. In addition, the purified cytosolic activator protein (of 80 k Da mass) for the H, K-ATPase system stimulates the gastric H, K-ATPase and the renal Na, K-ATPase to the same degree ^6^. These data revealed that the catalytic α-subunit (which faces cytosol) of the functional dual topology H, K-ATPase can also bind Na at an alkaline pH thus acting like a Na, K-ATPase. Such encouraging high pH neighboring the H, K-ATPase catalytic site would be attained during peak gastric acid secretion. This paper reviews the evidence pointing to the conclusion that the cytosolic regulation of the active transport of H/K, Na/K and Ca/H occurs in a tissue-specific manner where an individual P-2 ATPase subspecies is capable of transporting any of the other cations depending on the local ionic milleu in order to maintain optimal housekeeping conditions.

### Sole dependency of the apical membrane associated H, K-ATPase on the cytosolic endogenous activator (henceforth called “HAF”) for activity

HAF is an 80 k Da (a dimer of two identical 40 k Da subunits) cytosolic protein that occurs universally ^7– 13^ in the parietal cells. Activation of the gastric H, K-ATPase by the HAF is rather complex, resulting in a substantial increase in the affinity of the enzyme to K. Interaction of the HAF results in both up- and down-regulation of the gastric H, K-ATPase system depending on its concentration, showing a strong positive cooperativity (Hill coefficient = 4.5) followed by rapid decline ^9, 13^. The anti-HAF antibody completely blocks acid secretion in histamine-stimulated rabbit gastric glands demonstrating the essentiality of the HAF in gastric secretory process ^6^. Studies with phospholipase ^14^ and mild ethanol treatment ^15, 16^ revealed that the HAF dimer is rather loosely associated with the membrane-bound H, K-ATPase system, and the phospholipid is in some way involved in this process.

Such a loose association of the HAF with the secretory membrane of the parietal cell became clear when we studied the effects of HAF on the isolated apical (APM) and tubulovesicular (TV) membranes from rabbit gastric glands and observed characteristic differential effects ^17^. The APM showed very high basal (Ca or Mg-ATPase) activity with a negligible K-stimulated component (H, K-ATPase activity). When assayed with Mg, K and HAF, the K-stimulated component was greatly stimulated (over 100-fold) by the HAF ^17^. In contrast, the pure TV membranes exhibited a very low or negligible basal activity, but the very high K-stimulated ATPase activity only required a small amount of stimulation (only about 60%) by the HAF ^17^. These studies revealed that the HAF is not only loosely bound to the APM but also plays an essential role in gastric acid secretion thus supporting our earlier conclusion.

Such differential association between HAF and the APM and TV membranes was also reflected in their lipid profiles which were qualitatively similar but quantitatively very different. Thus, the phosphatidyl choline content of APM and TV was 67 and 33 µ moles/mg protein respectively with corresponding phosphatidyl choline/phosphatidyl ethanolamine ratios being 1.38 and 0.87 for APM and TV. Also, the phosphatidyl inositol and phosphatidyl serine content of APM were 24 and 8 µ moles/mg protein, respectively, about twice as much as that of TV ^17^. It may be noted that the APM and TV have different buoyant densities of 1.06 and 1.115 respectively, with a nearly equal phospholipid to cholesterol molar ratio of 0.64. The identity of APM was based on exclusive 5´-nucleotidase activity, unique vitamin B12 binding ability and characteristic quantitative differences in phospholipid make up from that of TV ^17^.

### Calcium (µM) regulation of the HAF dependent H, K-ATPase activity

During the activation of the H, K-ATPase system, the activator molecules demonstrate strong positive cooperativity (Hill coefficient = 4.5) followed by a rapid decline to zero suggesting the binding of the HAF with the H, K-ATPase oligomer occurs over a small activator concentration range ^9^. In other words, the bound HAF interacts in some way with the empty sites on the cytosolic domain of the H, K-ATPase to increase their affinity for the activator molecules. Similar to the sigmoidal activation and dramatic inhibition of the H, K-ATPase with increasing HAF, varying calcium concentrations (µM) also showed dual effects on the HAF-stimulated component of the H, K-ATPase. Low concentrations of calcium showed a small but consistent stimulation (about 20%) with a range of 0–1 µM followed by a dramatic inhibition abolishing the HAF-stimulated activity at 4 µM Ca ^17, 18^. Such positive cooperativity and down regulation with varying HAF and µM Ca concentrations are the marks of a delicate control mechanism inherent in the living system. The dramatic Ca-inhibition suggests a sequestration of Ca within the catalytic (cytosolic) domain of the gastric ATPase system. Such sequestration would depend primarily on the surface charge of the complex formed between the HAF and the H, K-ATPase catalytic domain and to a lesser extent on the nature of the neighboring phospholipid microdomain. This information suggests a combined role of calcium and cytosolic HAF in the intracellular regulation of gastric H transport.

It is obvious that an appropriate level of Ca (below 1 µM) facilitates a direct contact of the HAF with the catalytic surface of the enzyme while a higher concentration of Ca interferes presumably by building a critical barrier on the enzyme/HAF interface, thus preventing a direct interaction with the HAF. Under this condition, the apical membrane-located H, K-ATPase system would be acting as a provisional device for pumping out calcium prior to the onset of acid secretion ^17, 18^.

**Figure 1.**
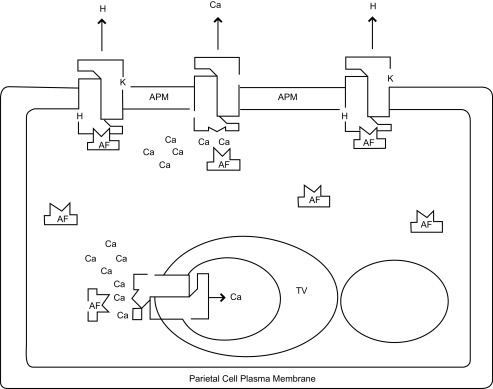
Critical interplay of calcium in the HAF-mediated (displayed as “AF”) regulation of the gastric H, K-ATPase pump showing oscillation between its H- and Ca-transporting modes depending on the local Ca level. In a similar fashion, the H, K-ATPase will also act as a Na-pump (not shown in the diagram) at the basolateral membrane depending on the local Na-concentration and pH. Following our current evidence, the critical interplay among the HAF, H, K-ATPase and Ca in parietal cells is depicted in this diagram. While the pump molecules integral to the tubulovesicle (TV) are stimulated appreciably by the HAF, those associated with the apical plasma membrane (APM) are absolutely dependent on the HAF for their function, revealing the essential nature of the HAF in gastric proton-pump function ^17^. For the ATPase assay ^9^ the desired amount of HAF (as indicated by the prior dose response study) was first pre-incubated with 5 µg of APM for 10 minutes at 37°C in 2 mM Pipes buffer (pH 7.4). The concentration of free Ca was regulated by varying Ca at a fixed concentration of 0.5 mM EGTA.

The suggested role of the APM-embedded H, K-ATPase as a provisional Ca-pump prior to acid secretion is fully consistent with recent reports from two different laboratories ^19– 21^. Using fluorescent-tagged antibodies against the plasma membrane Ca-ATPase (PMCA) and the gastric H, K-ATPase Caroppo *et al.* ^19^ demonstrated that not only do both ATPases have a closely similar and asymmetric distribution on the APM (of oxyntic cells of bullfrog gastric mucosa) but also were found to be co-localized by superimposition. At the same time, systematic studies with rabbit gastric glands by Michelangeli and coworkers ^20, 21^ revealed a consistent but transient peak of Ca transport into the secretary lumen prior to the onset of H-secretion. Such an oscillation between the two modalities of gastric H, K-ATPase system is depicted in Figure 1.

### Tissue origin and specificity of the HAF and the NaAF (activator specific for the Na, K-ATPase)

While the HAF is characteristically present in the parietal cells of the fundic mucosa, the NaAF was initially demonstrated in the cytosolic fractions of the brain and kidney from rabbits and also in pigs ^13^ and subsequently purified ^22^. A near homogeneous preparation of the NaAF, which has a mass of 170 k Da, was obtained by a modification of the procedure used for HAF purification ^22^. Unlike the 80 k Da HAF dimer, the NaAF is monomeric and has a 170 k Da mass in its active state. Also, the NaAF stimulates only the Na, K-ATPase without stimulating the H, K-ATPase, while the HAF is equally effective at stimulating both ^6^, suggesting that they share some domain(s) critical for the activation process.

In spite of such differences, some fundamental similarity was observed in the way HAF and NaAF work. Similar to the HAF-stimulated H, K-ATPase, the NaAF-stimulated Na, K-ATPase activity ^10, 22^ showed an abrupt inhibition within a relatively narrow range of Ca concentration. However, the HAF-stimulated H, K-ATPase activity was much more sensitive to Ca inhibition than the NaAF-stimulated Na, K-ATPase activity. The concentration of Ca needed for complete inhibition of the NaAF stimulation was 25–50 µM compared to the 3–4 µM for the HAF-stimulated H, K-ATPase ^10^.

It is noteworthy in this connection that the HAF has been found to possess high (NH _2_OH-insensitive) protein-kinase activity as demonstrated by its ability to phosphorylate histone, but at the same time the HAF is not auto-phosphorylated, and cannot be phosphorylated by heart protein kinase from Sigma ^13^. This rare ability to phosphorylate histone suggests that the HAF is capable of regulating its own intracellular level by regulating gene expression, thus raising the possibility of a similar intrinsic auto-regulation of the ubiquitous NaAF in a tissue specific manner. The existence of such auto-regulation of the NaAF would have great consequences in the metabolic and functional regulation of the cell as a whole.

### A model showing the gastric H, K-ATPase system acting in the altered modes

A model for the cytosolic regulation of the P-2 ATPase system in parietal cells by the HAF and µM Ca is depicted in Figure 1.

The pivotal roles of Ca in HAF regulation of the pump strongly imply that Ca acts as a physiological feedback control switch in gastric H transport ^10, 17^. The Figure also shows that in the presence of around 4 µM Ca, the Ca-inhibited H-pump spontaneously changes itself into a unique Ca-pumping mechanism for promptly reversing the Ca-induced inhibition, thus bringing the local calcium concentration back down to 1 µM at which point the HAF activation of the H-pump resumes. Such intimate interplay between Ca and HAF would also help the parietal cells to conserve energy by preventing the needless accumulation of H inside the cytosolic TV prior to their destined inclusion into the APM by subsequent fusion. This unique ability of >1 µM Ca concentrations to switch the inhibited gastric H-pump spontaneously to the Ca-pumping mode is also likely to be operative in other H-pumping epithelia such as the distal colon and kidney tubules ^23, 24^.

## Conclusion

The present paper proposes that the gastric H, K-ATPase, in addition to its well known role as a proton pump, may also act as a provisional Na-pump and a Ca-pump in the parietal cells where the HAF plays a critical role. Such altered modes demand immediate attention for a fresher look at the NaAF-regulated Na, K-ATPase system in various tissues. This is particularly critical for the central nervous system. The human brain, which weights only three pounds (about 2% of total body weight), consumes almost 25% of the total energy (ATP); thus it would be expected that the NaAF would play a major role in brain metabolism and function.
