# Supplementary material for: The parietal cell gastric H, K-ATPase also functions as the Na, K-ATPase and Ca-ATPase in altered states
Source: F1000Res. 2013 Sep 12;2:165. Originally published 2013 Jul 31. [Version 2] doi: 10.12688/f1000research.2-165.v2 (PMC3869522; doi:10.12688/f1000research.2-165.v2)
Supplement: Peer review history

# Peer review: Referee response for version 2

**Published:** 2013-09-16

**Authors:** Gabrielle Planelles

**Affiliations:** 1 Institut National de la Santé et de la Recherche Médicale (INSERM) UMRS-845, Paris, France

I have no further specific comment. I appreciate the change in title, as well as details that are now provided by the author. I think that this review may open an interesting field of scientific discussion.

I have read this submission. I believe that I have an appropriate level of expertise to confirm that it is of an acceptable scientific standard.

# Peer review: Referee response for version 1

**Published:** 2013-08-21

**Authors:** John Geibel

**Affiliations:** 1 School of Medicine, Yale University, New Haven, CT, USA

This commentary presents an interesting yet highly controversial view of acid secretion. The literature cited is very limited and is predominantly from the author’s previous work. 

My second point is that there are factual errors. There is no evidence for Calcium being pumped into the lumen of the gastric gland. The paper the author cites is measuring intracellular Ca not efflux of Ca into the lumen. In that study there are changes in intracellular Ca associated with carbachol that are typical for Ca activated H,K stimulated acid secretion. The critical studies have not been preformed that are necessary to prove the theory; namely block the H,K with omeprazole, stimulate the cells with increased intracellular Calcium (thapsigargin, carbachol, etc) and show measurements of luminal Ca concentration changes. 

As it stands now there are small pieces of unconnected data that do not give a convincing argument for this paper.

I have read this submission. I believe that I have an appropriate level of expertise to state that I do not consider it to be of an acceptable scientific standard, for reasons outlined above.

# Response

**Published:** 2013-09-13

**Authors:** Tushar Ray

**Affiliations:** RayIsles Inc., USA

The reason for such (seemingly odd) citations as pointed out by the reviewer is to clarify to the reader various important facets of the HAF in P-2 ATPase regulation within the parietal cells. To my knowledge no other laboratory came up with any publication either supporting or refuting our work so far which I could refer to. However, I will be most happy to be corrected by the reviewer if I am wrong.

Contrary to Dr. Giebel’s second statement on luminal Ca-transient, Caroppo et al ( EMBO J. 20, 6316-6326, 2001) directly measured the Ca-concentration using Ca-selective microelectrodes and reported the carbachol-induced Ca-transients in the gastric lumen.

# Peer review: Referee response for version 1

**Published:** 2013-08-21

**Authors:** Gabrielle Planelles

**Affiliations:** 1 Institut National de la Santé et de la Recherche Médicale (INSERM) UMRS-845, Paris, France

This review reflects the opinion that the gastric H,K-ATPase may transport other cations than potassium and protons, i.e., may transport sodium or calcium ions. 

According to the author, these different transport modes mostly depend on the local cytosolic composition (Na ^+^, pH, and Ca ^2+^), and on a cytosolic factor that acts in a dual manner on the pump activity. This interesting view of the mechanisms of gastric secretion is based on previous results that have been obtained by T. Ray's group. However, I would appreciate that the author further discuss the specificity of the reported assays, the purity of the preparations (apical and tubulovesicular membranes), and the possibility that other, physiologically quiescent, P-ATPases or channels may obscure the interpretation. 

Also, I feel that the title sounds different than the ms content, and that a change has to be considered.

I have read this submission. I believe that I have an appropriate level of expertise to confirm that it is of an acceptable scientific standard.

# Response

**Published:** 2013-09-13

**Authors:** Tushar Ray

**Affiliations:** RayIsles Inc., USA

Following the suggestions of Dr. Gabrielle Planelle the conditions used for monitoring the effects of HAF and µM Ca on gastric H, K-ATPase activity associated with the APM and TV have now been specified.  The purity and characteristic features of the isolated APM and TV membranes used in the study have also been provided. However, the possibility of interference under the conditions of our assay seems very unlikely. Also, following the excellent suggestion of the reviewer I have changed the title of the paper which now reads as, “The parietal cell gastric H, K-ATPase also functions as the Na, K-ATPase and Ca-ATPase in altered states”

# Peer review: Referee response for version 1

**Published:** 2013-08-05

**Authors:** Silvana Curci

**Affiliations:** 1 Department of Surgery, Harvard Medical School, MA, USA

Tushar Ray here provides a review of his work on the possible multiple roles of gastric H/K ATPase. 

Ray discusses the potentially interesting role of an endogenous activator of the H/K-ATPase, identified in the last few years by his research group.  Unfortunately I did not have the chance to read the recent study (submitted) where it was found that an “anti-activator antibody blocks acid secretion in histamine-stimulated glands”. Thus I am unable to really comment on this relevant point.

I was a bit confused by the title, expecting to read about “altered states” in which the H/K ATPase would assume multiple roles. Also it would be nice if the author could comment on the possibility that the function and activity of H/K ATPase and of the Na/K ATPase would vary depending on the specific location of the parietal cells in the gland (i.e. more luminal or more basal), see Fujii et al. (2008) (JBC 2008. 283, 6869-6877).

Minor comments: a couple of typos at page 3: 2nd paragraph “of the functional dual topology H,K ATPase”;  4th paragraph “The APM showed very high basal( Ca2+….)”.

I have read this submission. I believe that I have an appropriate level of expertise to confirm that it is of an acceptable scientific standard.

# Response

**Published:** 2013-09-13

**Authors:** Tushar Ray

**Affiliations:** RayIsles Inc., USA

We studied the role of the HAF in proton production using a monospecific anti-HAF antibody raised in female rabbits against a pure preparation of the HAF.  The histamine-stimulated acid secretion (as measured by 14C-aminopyrene uptake) in isolated rabbit gastric glands was eliminated by the anti-HAF-antibody proving the essential role of the HAF in the production of protons at the catalytic domain inside parietal cells (DOI: 10.5281/zenodo.7093). Following the excellent suggestion of Dr. Silvana Curci, I have changed the title which now reads as. “ **The parietal cell gastric H, K-ATPase also functions as the Na, K-ATPase and Ca-ATPase in altered states”**

The issue of variable gastric acid secretion depending on location of the parietal cells in a gland is an important one, and I thank the reviewer for raising this issue.  Since the parietal cells at the base of the gland secrete very little acid and are known to remain practically unaffected by gastric ulcers, one would expect to see a marked reduction both in the total number of H-pumps as well as in the turnover of the existing pumps.  Also, one would expect to see a proportionate reduction in the altered function of the H, K-ATPase as provisional Na-pumps in these base glandular cells. Since the activity and function of both H- and Na-pumps rely on the cytosolic HAF, a noticeable change is expected to occur at the level of HAF in these cells. Thus a comparison in the activity and turnover of the HAF in parietal cells nearest to the secretary canneliculi of gastric glands compared to those farthest at the glandular base needs to be investigated.

Pondering over the turnover of HAF in parietal cells I recalled some novelty of the HAF which now appears relevant.  The HAF was previously demonstrated to possess high (NH2OH-insensitive) protein-kinase activity by its ability to phosphorylate histone, but at the same time HAF is not auto-phosphorylated, and cannot be phosphorylated by heart protein kinase from Sigma (reference 13, DOI: 10.5281/zenodo.7095 ). This rare ability to phosphorylate histone suggests that the HAF is capable of regulating its own intracellular level by directing gene expression, thus raising the possibility of a similar auto-regulation by the NaAF in other cell types. This aspect has now been discussed in the revised version.
